# Neoadjuvant and Adjuvant Immunotherapy in Early-Stage Non-Small-Cell Lung Cancer, Past, Present, and Future

**DOI:** 10.3390/jcm10235614

**Published:** 2021-11-29

**Authors:** Chun Ho Szeto, Walid Shalata, Alexander Yakobson, Abed Agbarya

**Affiliations:** 1 Medical School for International Health, Ben-Gurion University of the Negev, Beer Sheva 84101, Israel; szeto@post.bgu.ac.il; 2 The Legacy Heritage Oncology Center & Dr. Larry Norton Institute, Soroka Medical Center & Ben-Gurion University, Beer Sheva 84105, Israel; walid_sh@clalit.org.il (W.S.); alexy@clalit.org.il (A.Y.); 3 Oncology Department, Bnai Zion Medical Centre, Haifa 31048, Israel

**Keywords:** adjuvant, immune checkpoint inhibitors, immunotherapy, neoadjuvant, non-small-cell lung cancer (NSCLC)

## Abstract

Lung cancer is worldwide the most common malignancy. Standard of care treatments for early-stage non-small-cell lung cancer (NSCLC) include surgery and adjuvant chemotherapy. However, these patients continue to have poor prognosis due to systemic or local relapse. Immunotherapy has been considered as a novel approach to improve survival in patients with early-stage NSCLC. Since immune checkpoint inhibitors have transformed the treatment of advanced NSCLC, there is a growing interest in the role of immunotherapy in early-stage NSCLC. In this review, we summarize reported and ongoing clinical trials of immunotherapy in both neoadjuvant and adjuvant settings. We also highlight unaddressed issues in this field of research, such as the predictive markers, the optimal combination therapy, and the need for adjuvant immunotherapy. More studies are needed to optimize the treatment regimen of immunotherapy in patients with early-stage NSCLC.

## 1. Introduction

Lung cancer is the leading cause of cancer death globally, with an estimated 1.8 million deaths in 2020 [1]. Non-small-cell lung cancer (NSCLC) is the most common type of lung cancer, accounting for 85% of all lung cancer diagnoses [2]. Histologically, NSCLC is divided into three main types: adenocarcinoma, squamous cell carcinoma, and large cell carcinoma [3]. NSCLC is often insidious and undiagnosed until advanced-stage disease is present [4]. Approximately 25% of patients with NSCLC have localized disease at the time of diagnosis [5]. Lobectomy followed by systemic adjuvant therapy is considered as standard treatment for patients with resectable NSCLC. Despite the standard treatment, 50% of patients with stage II, and 60% of patients with stage IIIA disease die within five years [6]. Therefore, researchers have been exploring novel treatment approaches to reduce the risk of recurrence and improve survival of resectable NSCLC.

Adjuvant chemotherapy, comprising of cisplatin-based combination regimen, is a current standard of care in stage II and IIIA NSCLC after surgical resection [7]. The idea behind the use of adjuvant treatment for early-stage NSCLC is to treat micrometastatic disease and prevent recurrence. A meta-analysis found that adjuvant chemotherapy gave an absolute survival improvement of 4% at 5 years, compared to surgery alone, to patients with resected early-stage NSCLC [8]. The benefit from adjuvant chemotherapy is modest yet consistent. 

Neoadjuvant treatment is considered as another approach to improve survival in patients with resectable NSCLC. Neoadjuvant treatment offers several potential advantages, including downstaging of the tumor, which allows complete resection; early treatment of micrometastases; and improved tolerability. However, the use of neoadjuvant chemotherapy for resectable NSCLC is still debatable. A meta-analysis found an absolute 5% survival benefit at 5 years with preoperative chemotherapy, compared to surgery alone, among patients with stage IB to IIIA NSCLC [9]. That being said, a systematic review of 32 randomized trials showed that there was no difference in survival between preoperative and postoperative chemotherapy [10]. Thus, although neoadjuvant chemotherapy improves survival compared to surgery alone, it does not provide extra benefit over surgery followed by adjuvant chemotherapy for the treatment of resectable NSCLC. 

Current immune checkpoint inhibitors (ICI) therapy works by blocking the interaction between programmed death-ligand 1 (PD-L1) on tumor cells and programmed cell death protein 1 (PD-1) on T cells, thereby allowing the T cells to kill tumor cells. Recent studies have proved that ICI monotherapy and in combination with chemotherapy improved survival, with manageable toxicity, in a subset of advanced NSCLC patients [11,12]. There is a growing interest in evaluating the efficacy and safety of ICI in patients with early-stage NSCLC. In this review paper, we examine past and ongoing clinical trials on neoadjuvant and adjuvant immunotherapies for patients with resectable NSCLC.

## 2. Materials and Methods

Multiple searches were run in PubMed and ClinicalTrials.gov from inception to September 2021 for clinical trials that included neoadjuvant or adjuvant ICI in resectable NSCLC. Search terms included “early-stage NSCLC”, “neoadjuvant immunotherapy”, “adjuvant immunotherapy". All completed trials with available results are included in our review. Ongoing trials were selected based on its phase and the treatment used. All ongoing phase 3 trials are included. Ongoing trials that tested novel ICIs are also included. 

## 3. Past Clinical Trials That Included Only Neoadjuvant Immunotherapy in Patients with Resectable NSCLC 

### 3.1. ChiCTR-OIC-17013726

This study was a single-center phase 1b trial registered in China. Forty treatment-naïve patients with resectable NSCLC (stage IA–IIIB) were enrolled to this study [13]. All participants received two cycles of sintilimab, and 37 underwent surgical resection afterwards. Of these 37 participants, 40.5% achieved a major pathologic response (MPR), including 16.2% with a pathologic complete response (pCR) in primary tumor and 8.1% in lymph nodes. Interestingly, squamous cell NSCLC patients showed superior response compared with patients bearing NSCLC adenocarcinoma (MPR: 48.4% versus 0%, respectively). Out of all enrolled patients, 10% experienced grade 3 or higher treatment-related adverse events (TRAE); the most common one was pneumonitis (Table 1).

### 3.2. NA_00092076 (NCT02259621)

In this study, 22 patients with surgically resectable NSCLC (stage I to IIIA) were enrolled from two centers in the United States [14]. Prior to surgery, participants received up to two cycles of nivolumab. Twenty patients received two doses of nivolumab, had their tumors completely removed, and demonstrated 45% MPR. Five of 22 patients had TRAE of any grades, and only one event was grade 3 or higher.

Based on these results, the investigators expanded this study to include an arm of neoadjuvant nivolumab plus ipilimumab [15]. Nine patients with resectable NSCLC (stage IB to IIIA) were enrolled and given three cycles of nivolumab and a single dose of ipilimumab prior to surgery. All patients completed the scheduled neoadjuvant treatment and were fit for surgery without treatment-related delays; however, six of nine patients (67%) experienced TRAE, with three (33%) having grade ≥3 TRAE, including one case of acute respiratory distress syndrome (grade 5). Other grade 3 TRAE included pneumonitis, rash, pruritus, and headache. Of six patients who underwent surgery, two (33%) achieved a pCR. Due to the toxicity, this study arm of neoadjuvant nivolumab plus ipilimumab was terminated early (Table 1).

### 3.3. TOP1501 (NCT02818920)

Thirty patients with untreated resectable NSCLC (stage IB–IIIA) were enrolled in this multi-institutional phase 2 study [16]. Prior to surgery, enrolled patients were given two cycles of pembrolizumab. In total, 25 patients underwent surgical resection, and 17 patients received four cycles of adjuvant pembrolizumab after completing standard adjuvant therapy. Among the patients who completed surgery, 28% demonstrated an MPR. The most common TRAE was diarrhea, which was followed by fatigue and rash. There was only one grade 3 adverse event (psoriasis flare) (Table 1).

### 3.4. LCMC3 (NCT02927301)

The LCMC3 study recruited 181 patients with untreated resectable stage IB–IIIB NSCLC from 21 centers in the United States [17]. Participants received two cycles of neoadjuvant atezolizumab prior to surgery; those participants who demonstrated clinical benefit were eligible to receive up to 12 months of adjuvant atezolizumab. Following neoadjuvant atezolizumab, unresectability was detected in 29 patients. In participants without epidermal growth factor receptor (EGFR) or anaplastic lymphoma kinase (ALK) mutations who completed surgery, 20% achieved an MPR and 7% achieved a pCR. Only 5% of enrolled patients experienced grade 3 or higher TRAE prior to surgery. Detailed results will be reported in the future. 

### 3.5. IONESCO (NCT03030131)

Forty-six eligible patients with resectable NSCLC (stage IB–IIIA, non N2) were recruited to the IONESCO trial [18]. Participants received three courses of durvalumab, which was followed by surgical resection. The primary endpoint was percentage of complete surgical resection, while the secondary endpoints included safety, overall survival (OS), disease-free survival (DFS), and MPR. The IONESCO trial was terminated because of an excess in 90-day postoperative mortality (four deaths, 9%). There was no grade 3 or higher TRAE. A detailed report will be presented in the future (Table 1).

### 3.6. NEOSTAR (NCT03158129)

In the NEOSTAR trial, 44 patients with operable NSCLC were recruited, 23 were randomized to nivolumab monotherapy, and 21 patients were randomized to a combination of nivolumab + ipilimumab [19]. In the nivolumab arm, participants were given three cycles of nivolumab, and in the dual therapy arm, patients were given three cycles of nivolumab plus a dose of ipilimumab in the first cycle. After completing the treatments, patients in both arms underwent surgical resection of the primary tumor with lymphadenectomy. Most (96%) of the patients in the nivolumab arm and 90% in the dual therapy arm completed the planned neoadjuvant therapy regimen. In total, 22% of patients in the nivolumab arm and 38% of patients in the dual therapy arm achieved an MPR in the intention-to-treat population. pCR was observed in two patients after nivolumab monotherapy and in six patients treated with combined therapy of nivolumab and ipilimumab. Grade 3–5 TRAE were reported in 13% of patients in the nivolumab arm and 10% of patients in the dual therapy arm (Table 1).

## 4. Past Clinical Trials That Included Neoadjuvant with a Combination of Chemoimmunotherapy in Patients with Resectable NSCLC 

### 4.1. SAKK 16/14 (NCT02572843)

In the SAKK 16/14 trial, 68 patients with stage IIIA(N2) NSCLC were enrolled from 14 sites in Switzerland [20]. Prior to surgery, participants received three cycles of neoadjuvant chemotherapy (cisplatin and docetaxel) once every 3 weeks, which was followed by two doses of durvalumab once every 2 weeks. Adjuvant durvalumab was continued for one year after surgery. Fifty-five patients underwent surgical resection of NSCLC; of them, 34 patients (62%) achieved an MPR, and 10 patients achieved a complete pathologic response. The 1-year event-free survival (EFS) rate was 73% (two-sided 90% CI, 63 to 82). In total, 59 patients (88%) had a grade ≥ 3 adverse event. The most common TRAE were elevated alanine aminotransferase (ALT), dyspnea, fatigue, and lung infection. One grade 4 treatment related elevated lipase level was observed. There was no grade 5 TRAE (Table 2).

### 4.2. NCT02716038

The NCT02716038 recruited 30 patients with resectable stage IB–IIIA NSCLC from three hospitals in the USA [21]. Participants received a combined neoadjuvant treatment of atezolizumab, carboplatin, and nab-paclitaxel. Patients without disease progression after two cycles of treatment would proceed to receive two additional cycles prior to surgical resection. In total, 26 patients underwent successful surgical resection. Of all enrolled patients, 57% had an MPR, while 33% had a pCR. The most common treatment related grade 3–4 adverse events included neutropenia, thrombocytopenia, increased aspartate aminotransferase (AST) concentrations, and increased ALT concentration (Table 2).

### 4.3. NADIM (NCT03081689)

The NADIM trial reported the participation of 46 patients from 18 hospitals in Spain [22]. The study included patients with treatment-naïve NSCLC of stage IIIA that was considered surgically resectable. The patients were assigned to a single-arm combined treatment of nivolumab, carboplatin plus paclitaxel for three cycles before surgical resection, followed by adjuvant nivolumab monotherapy for one year. Forty-one patients had surgery. The progression-free survival (PFS) was 77.1% (95% CI 59.9–87.7) at 24 months. In total, 93% of the patients experienced TRAE during neoadjuvant treatment, of which 30% accounted for grade 3 or worse. The most common grade 3 or worse TRAE were increased lipase and febrile neutropenia. Other grade 3 adverse events included neurotoxicity and increased levels of serum amylase and creatinine (Table 2).

### 4.4. NeoTAP01 (NCT04304248)

In the NeoTAP01 trial, 33 patients with deemed resectable stage III NSCLC were enrolled from a single center in China [23]. All participants were given three cycles of neoadjuvant treatment of toripalimab, carboplatin, and pemetrexed or nab-paclitaxel. After completing the neoadjuvant treatment, 30 participants underwent surgical resection. Of these 30 patients, 20 (66.7%) achieved an MPR, and 15 (50%) achieved a complete pathologic response. The most common grade 3–4 TRAE was anemia. There was one severe TRAE (peripheral neuropathy (Guillain–Barré syndrome), grade 3). No grade 4–5 TRAE was observed (Table 2). 

## 5. Past Clinical Trials That Included Only Adjuvant Immunotherapy in Patients with Resected NSCLC 

### 5.1. MACRIT (NCT00480025)

In total, 12,820 patients, from 443 centers across 43 countries, were recruited to this study [24]. All patients had stage I to IIIA MAGE-A3 expressing NSCLC. Prior to enrolling in the trial, the patients had the NSCLC resected, with or without subsequent adjuvant chemotherapy. The patients were randomized and assigned to two arms: the first arm in which the patients received up to 13 intramuscular injections of recMAGE-A3 with AS15 immunostimulant (MAGE-A3 immunotherapeutic) for 27 months, and the second arm in which patients received placebo for 27 months. Compared to placebo, adjuvant treatment with MAGE-A3 immunotherapeutic did not improve DFS in patients with MAGE-A3-positive surgically resected NSCLC (hazard ratio (HR) 1.02, 95% confidence interval (CI) 0.89–1.18; *p* = 0.74). As the survival benefits of MAGE-A3 immunotherapeutic for NSCLC patients were not significant, its use for treating NSCLC has stopped. Both arms expressed similar frequency of grade 3 or worse adverse events such as infection/infestation, vascular disorders, and neoplasm.

### 5.2. IMpower 010 (NCT02486718)

A total of 1280 patients, from 227 sites in 22 countries, were enrolled in this open-label phase III study [25]. All patients had completely resected NSCLC (stage IB–IIIA). After completing up to four cycles of adjuvant platinum-based chemotherapy, eligible participants were randomized to receive atezolizumab for 16 cycles or best supportive care. Compared with best supportive care, adjuvant atezolizumab significantly improved DFS in all patients in the stage II–IIIA population (HR 0.79, 95% CI 0.64–0.96; *p* = 0.020), in the stage II–IIIA patients group whose tumors expressed PD-L1 ≥ 1% (HR 0.66, 95% CI 0.5–0.88; *p* = 0.0039) and in the intention-to-treat population (HR 0.81, 95% CI 0.67–0.99; *p* = 0.040). In total, 11% of the patients in the treatment arm experienced grade 3 and 4 atezolizumab-related adverse events, and 1% of the patients had grade 5 events. In the atezolizumab group, the most commonly reported grade 3–4 adverse events were pyrexia (1%) and increased levels of ALT (2%) and AST (1%).

## 6. Selected Ongoing Trials That Included Neoadjuvant Immunotherapy in Patients with Resectable NSCLC

### 6.1. MK3475-223 (NCT02938624)

MK3475-223 is a phase I dose escalation trial of neoadjuvant pembrolizumab in resectable NSCLC (stage I–II) [26]. Participants receive neoadjuvant pembrolizumab prior to surgery. When three subjects in a dose cohort completed the dose-limiting toxicity evaluation period, dosing at the next higher level was initiated. The primary outcome measures of this study include dose-limiting toxicity, percentage of residual viable tumor cells, and percent change in tumor volume. The study was estimated to be completed in April 2021 (Table 3).

### 6.2. NA_00092076 (NCT02259621)

Currently, the NA_00092076 study is recruiting patients with treatment-naïve resectable NSCLC for its third study arm [14,15]. In this study arm, enrolled patients are given three cycles of nivolumab plus platinum doublet chemotherapy. Safety is the primary endpoint, while feasibility and pathologic response are the key secondary endpoints. The estimated study completion date is in January 2023 (Table 3).

### 6.3. NEOSTAR (NCT03158129)

The NEOSTAR trial is recruiting patients into two arms, in which eligible patients are randomized and treated with neoadjuvant chemotherapy, combined with either nivolumab monotherapy or dual therapy of nivolumab plus ipilimumab [19]. The primary endpoint is MPR. The estimated completion date of NEOSTAR is July 2022 (Table 3).

### 6.4. CANOPY N (NCT03968419)

CANOPY N is an open-label phase II trial planning to recruit 110 patients with resectable NSCLC (stage IB–IIIA) [27]. Eligible participants are randomized 2:2:1 to canakinumab once every 3 weeks monotherapy or in combination with pembrolizumab once every 3 weeks or pembrolizumab monotherapy once every 3 weeks. Participants are treated for up to two cycles and then submitted to surgery. The primary endpoint of this study is MPR rate. Secondary outcome measures include antidrug antibodies of canakinumab and pembrolizumab, overall response rate, and surgical feasibility rate. The estimated study completion date is March 2023 (Table 3).

### 6.5. NCT04379739

NCT04379739 is a single-center open-label phase II study [28]. Approximately 82 patients with resectable NSCLC (stage II–IIIA) will be enrolled and assigned to receive camrelizumab with either apatinib or platinum-based chemotherapy for up to four cycles, which is followed by surgical resection. The primary outcome measure is MPR rate. Secondary outcome measures include OS, DFS, and safety. The study is expected to be completed in December 2026 (Table 3).

### 6.6. NCT04560686

This NCT04560686 single-arm phase II study has an estimated enrollment of 23 patients with stage I–IIIB NSCLC [29]. Eligible patients are given three cycles of bintrafusp alfa prior to surgery. The primary endpoint is pCR rate. Secondary endpoints include OS, incidence of adverse events, and pCR. The study is expected to be completed in October 2024 (Table 3).

### 6.7. CheckMate 816 (NCT02998528)

CheckMate 816 is a multinational, open-label phase III trial with an estimated enrolment of 350 patients with resectable NSCLC (stage IB–IIIA) [30]. Eligible participants are randomized to the following three neoadjuvant therapies prior to surgery: platinum doublet chemotherapy vs. nivolumab plus platinum doublet chemotherapy vs. nivolumab plus ipilimumab. The primary outcome measures are EFS and pCR rate. Secondary outcome measures include OS, MPR rate, and time to death or distant metastases. The study is expected to be completed in November 2028 (Table 3).

### 6.8. KEYNOTE 671 (NCT03425643) 

KEYNOTE 671 is a double-blind phase III trial with an estimated enrolment of 786 patients with resectable NSCLC (stage II to IIIB) [31]. Eligible participants are randomized to neoadjuvant pembrolizumab vs. placebo, in combination with platinum doublet neoadjuvant chemotherapy. After surgical resection, patients received adjuvant pembrolizumab or placebo. The primary outcome measures of this study include EFS and OS. Secondary outcome measures include MPR rate, pCR rate, global health status/quality of life score, adverse events, perioperative complications, and treatment discontinuations due to adverse events. The estimated completion date of this study is June 2026 (Table 3).

### 6.9. IMpower 030 (NCT03456063)

IMpower 030 is a double-blind phase III trial that has recruited 453 patients with resectable NSCLC (stage II, IIIA, and selected IIIB (T3N2only)) [32]. Prior to surgery, eligible participants are randomized to atezolizumab plus platinum-based chemotherapy vs. placebo plus platinum-based chemotherapy. The primary endpoint is independent review facility-assessed EFS. Secondary endpoints include pCR, MPR, OS, and DFS. The estimated study completion date is April 2026 (Table 3).

### 6.10. AEGEAN (NCT03800134)

AEGEAN is a double-blind phase III trial [33]. Approximately 800 patients with resectable NSCLC (stage II–III) will be enrolled and randomized 1:1 to receive either durvalumab or placebo every 3 weeks alongside platinum-based chemotherapy prior to surgery, followed by either adjuvant durvalumab or placebo every 4 weeks for additional 12 cycles post-surgery. The primary outcome measures include pCR in modified intent-to-treat and EFS. Secondary outcome measures include DFS, MPR, and OS. The study is expected to be completed in April 2024 (Table 3).

### 6.11. CheckMate 77T (NCT04025879)

CheckMate 77T (NCT04025879) is a multinational, double-blind phase III trial [34]. Approximately 452 patients with resectable stage IIA–IIIB NSCLC will be enrolled and randomized to receive nivolumab or placebo alongside with carboplatin- or cisplatin-based doublet chemo, which will be followed by surgery. Adjuvant treatment with nivolumab or placebo will be given to participants who undergo surgical resection. The primary endpoint is EFS. Secondary endpoints include OS, MPR, complete pathologic response, and safety. The estimated completion date is September 2024 (Table 3).

## 7. Ongoing Trials That Included Adjuvant Immunotherapy in Patients with Resected NSCLC

### 7.1. BR.31 (NCT02273375)

The BR.31 trial is a double-blind phase III trial that recruited patients with completely resected NSCLC (stage IB–IIIA) [35]. A total of 1360 participants are estimated to be enrolled. After receiving adjuvant chemotherapy, participants are randomized to receive either durvalumab or placebo every 2 weeks for a year. The primary outcome measures include overall DFS and DFS in PD-L1 positive participants. The study completion date is estimated to be January 2024 (Table 4).

### 7.2. PEARLS (NCT02504372)

The Pearls trial has recruited 1177 patients with resected NSCLC (stage IB–IIIA) [36]. After completing standard adjuvant therapy, participants are randomized to receive adjuvant pembrolizumab vs. placebo every 3 weeks for a year. The primary endpoints of the study are DFS in the PD-L1 strong positive subgroup and in the overall population, while secondary endpoints include DFS in the PD-L1 positive subgroup, OS in each subpopulation and in the overall population, lung cancer-specific survival, and safety and tolerability. The study completion date is estimated to be February 2024 (Table 4).

### 7.3. ANVIL (NCT02595944)

In this open-label study, 903 patients with completely resected NSCLC (stage IB to IIIA NSCLC without EGFR or ALK mutations) are estimated to be enrolled [37]. Patients who had adjuvant chemotherapy and/or radiotherapy are allowed to join the study. Eligible participants are randomized to adjuvant nivolumab (every four weeks, up to one year) versus standard of care observation. The primary outcome measures include OS, DFS, and DFS in NSCLC with high PD-L1 expression. The estimated primary completion date is July 2024 (Table 4).

### 7.4. CANOPY-A (NCT03447769)

A total of 1500 participants with resected NSCLC (stage IIA–IIIA, IIIB with N2 disease only) are estimated to be enrolled [38]. After completing standard-of-care adjuvant treatments, participants are randomized 1:1 to receive adjuvant canakinumab vs. placebo for up to 18 cycles. The primary endpoint of the study is DFS assessed by local investigators, and secondary endpoints includes OS, lung cancer-specific survival, safety, pharmacokinetics and immunogenicity of canakinumab, and patient-reported outcomes. The estimated completion date is January 2027 (Table 4).

### 7.5. MERMAID-1 (NCT04385368)

MERMAID-1 is a phase III, multicenter, double-blind study that will recruit approximately 332 minimal residual disease (MRD) positive patients with completely resected stage II–III NSCLC [39]. Participants are randomized to receive adjuvant durvalumab plus standard of care chemotherapy versus placebo plus standard of care immunotherapy. The primary endpoint of this study is DFS in the MRD positive analysis set. Secondary endpoints include DFS in the full analysis set (investigator-assessed and blinded independent central review); DFS in the MRD positive analysis set (blinded independent central review); OS in the MRD positive analysis set and the full analysis set; safety and tolerability; and patient-reported outcomes. The estimated study completion date is September 2026 (Table 4).

### 7.6. MERMAID-2 (NCT04642469)

MERMAID-2 is a phase III, multicenter, double-blind study [40]. Patients who have completely resected stage II–III NSCLC will be enrolled and monitored regularly for MRD emergence via analyzing circulating tumor DNA level in plasma samples. Approximately 284 MRD+ patients will be randomized 1:1 to receive adjuvant durvalumab versus placebo every 4 weeks for up to 24 months. The primary endpoint is DFS in patients with PD-L1 tumor cell expression ≥ 1%. Secondary endpoints include DFS in the full analysis set, PFS, OS, time to subsequent therapy, patient-reported outcomes, and safety. The estimated study completion date is October 2027 (Table 4).

## 8. Discussion

Early-stage NSCLC has an unsatisfactory prognosis due to systemic or local relapse, despite the current stand of care treatment with curative resection followed by adjuvant chemotherapy [6]. Recent development of novel therapeutic approaches, such as tyrosine kinase inhibitors (TKI), has changed the management of patients with certain subtypes of NSCLC. The ADAURA study showed that osimertinib, a third-generation EGFR-TKI, markedly improved DFS, with tolerable toxicity, in patients with completely resected EGFR mutant NSCLC [41]. Therefore, osimertinib has been approved for adjuvant treatment for patients with resected NSCLC harboring EGFR mutations [42]. Although EGFR-TKI is an effective treatment, EGFR mutation can only be identified in 14.1% to 38.4% of all NSCLC [43]. Adjuvant chemotherapy remains to be the mainstay of postoperative treatment for patients with early-stage non-EGFR mutant NSCLC.

Immunotherapy is another approach for improving long-term survival for patients with NSCLC. Immunotherapy, ICI in particular, has become a part of standard of care treatment for patients with advanced NSCLC [44,45,46]. In the setting of early-stage NSCLC, the first reported phase 3 trial of adjuvant immunotherapy for early-stage NSCLC, MACRIT, had a negative result [24]. However, a recently published study, IMpower 010, showed that adjuvant atezolizumab significantly improved survival after adjuvant chemotherapy in patients with resected NSCLC compared to best supportive care [25]. Thus, adjuvant atezolizumab, following resection and platinum-based chemotherapy, has been approved for patients with stage II to IIIA NSCLC [47]. Data from other ongoing phase 3 adjuvant studies (PEARLS, BR31, ANVIL, MERMAID-1, and MERMAID-2) might further elucidate the role of ICIs in the adjuvant setting in resected NSCLC. In addition to ICI, the efficacy and safety of adjuvant canakinumab, an interleukin-1β blocker, is being evaluated in a phase 3 trial (CANOPY-A).

Similar to TKI, immunotherapy does not work for everyone. It is important to define the patients who will most benefit from ICI. PD-L1 expression and tumor mutational burden (TMB) have been used to predict the efficacy of various ICIs in patients with advanced NSCLC. In the adjuvant setting, IMpower 010 showed a certain degree of correlation between DFS and PD-L1 expression level [25]. Nevertheless, in the neoadjuvant setting, trials have shown contradictory results. PD-L1 expression was significantly associated with MPR rate in NEOSTAR but not in LCMC3, NA_00092076, NADIM, NeoTAP01, and NCT02716038 [17,19,20,21,22,23]. Conversely, TMB was associated with MPR in NA_00092076 but not in LCMC3. There are other emerging biomarkers, such as tumor-infiltrating lymphocytes, circulating tumor DNA, and specific molecular alterations. However, evidence regarding their predictive value in the neoadjuvant or adjuvant setting is still scarce. Most ongoing trials assess only PD-L1 but no other potential biomarkers. MERMAID-1 and -2 are studies of note because they assess the benefits of adjuvant durvalumab in patients with MRD + status, which is determined by circulating tumor DNA [39,40]. The identification of optimal biomarkers is of utmost importance, as giving immunotherapy to non-responding patients may delay curative resection and increase the risk of progressive disease. Further analysis of these biomarkers is needed to evaluate their predictive utility for immunotherapy in early-stage NSCLC.

Combination therapy is common for patients with cancer. In addition to chemoimmunotherapy, nivolumab plus ipilimumab is another combination of note for NSCLC. In the Checkmate 227 trial, treatment with nivolumab plus ipilimumab significantly improved OS in patients with advanced NSCLC [48]. In the neoadjuvant setting, NEOSTAR and NA_00092076 are two phase 2 studies in which nivolumab, with or without ipilimumab as the treatment regimen, was administered in patients with resectable NSCLC. Both studies showed improved pCR rate on the nivolumab plus ipilimumab arm compared to the nivolumab arm [15,19]. The results may suggest that combination immunotherapy is superior to single-agent immunotherapy in treating resectable NSCLC. Nonetheless, the sample sizes of NEOSTAR and NA_00092076 were small. The treatment arm of nivolumab plus ipilimumab in NA_00092076 was prematurely aborted due to toxicity. Larger phase 3 studies are needed to confirm the efficacy of this combination immunotherapy for early-stage NSCLC. Other combinations of treatments should be explored in the future as well.

Current phase 3 trials in early-stage NSCLC are conducted in similar settings. All neoadjuvant studies compare chemoimmunotherapy with chemotherapy, and most adjuvant studies assess the efficacy of immunotherapy compared with placebo or best of care. Among the neoadjuvant studies, CheckMate 816 is the only trial that has an additional arm of combination immunotherapy (nivolumab and ipilimumab). There is no phase 3 trial comparing adjuvant immunotherapy or chemoimmunotherapy with neoadjuvant immunotherapy or chemoimmunotherapy, or comparing single-agent immunotherapy with chemoimmunotherapy. Since more evidence has revealed the benefit of immunotherapy, there is a need to compare different treatment options and dosages in order to optimize the treatment regimen of immunotherapy in early-stage NSCLC (Table 5 and Table 6).

## 9. Conclusions

Immunotherapy and targeted therapy have revolutionized the treatment of NSCLC in the adjuvant setting. In the neoadjuvant setting, reported phase II trials showed positive yet preliminary results. Ongoing trials may shed light on the safety and efficacy of other immunotherapeutic agents in patients with early-stage NSCLC. Questions regarding the predictive biomarkers, the optimal combination therapy, and the need for adjuvant immunotherapy remain to be answered. More studies are needed to address these issues and to achieve precision medicine in the field of NSCLC treatment.

## Figures and Tables

**Table 1:** Past clinical trials that included neoadjuvant immunotherapy in resectable NSCLC.

NCT Number	Name	Phase	Stage	Participants (*n*)	Treatment Arm(s)	Primary Endpoint	MPR (%)	pCR (%)	Common Grade ≥ 3 TRAE
**ChiCTR-OIC-17013726**		1b	IA–IIIB	40	Sintilimab	Safety	40.5 *	16.2 *	Pneumonitis (5%)
**NCT02259621**	NA_00092076	2	IB–IIIA	22 9	1. Nivolumab 2. Nivolumab + ipilimumab	Safety	45 * 33 *	15 * 33 *	Pneumonia (5%) Rash (11%), Pruritus (11%), ARDS (11%), Headache (11%), Pneumonitis (11%)
**NCT02818920**	TOP1501	2	IB–IIIA	30	Pembrolizumab	Surgical feasibility rate	28 *	12 *	Psoriasis flare (3%)
**NCT02927301**	LCMC3	2	IB–IIIB	181	Atezolizumab	MPR	20 ***	7 ***	TBR
**NCT03030131**	IONESCO	2	IB–IIIA	46	Durvalumab	Percentage of complete surgical resection	TBR	TBR	No grade ≥ 3 TRAE
**NCT03158129**	NEOSTAR	2	IA–IIIA	23	1. Nivolumab	MPR	22 **	9 **	Hypermagnesemia (4%), hypoxia (4%), pneumonia (4%), pneumonitis (4%)
				21	2. Nivolumab + ipilimumab		38 **	29 **	Diarrhea (5%), hyponatremia (5%)

Abbreviations: MPR, major pathologic response; pCR, pathologic complete response; TRAE, treatment-related adverse events; ARDS, acute respiratory distress syndrome; TBR, to be reported * denominator = number of patients who underwent surgical resection; ** denominator = intention-to-treat population; *** denominator = number of patients without EGFR and ALK mutations who underwent surgical resection.

**Table 2:** Past clinical trials that included neoadjuvant chemoimmunotherapy in resectable NSCLC.

NCT Number	Name	Phase	Stage	Participants(*n*)	Treatment Arm(s)	Primary Endpoint	MPR (%)	pCR (%)	Common Grade ≥ 3 TRAE
**NCT02572843**	SAKK 16/14	2	IIIA	68	Durvalumab + cisplatin + docetaxel	EFS	62 *	18 *	Increased ALT (5%), dyspnea (5%), fatigue (5%), lung infection (5%)
**NCT02716038**		2	IB–IIIA	30	Atezolizumab + carboplatin + nab-paclitaxel	MPR	57 **	33 **	Neutropenia (50%), thrombocytopenia (7%), increased AST (7%), increased ALT (7%)
**NCT03081689**	NADIM	2	IIIA	46	Nivolumab + carboplatin + paclitaxel	PFS	83 *	63 *	Increased lipase (7%), febrile neutropenia (7%)
**NCT04304248**	NeoTAP01	2	III	33	Toripalimab + carboplatin + (nab-bound paclitaxel or pemetrexed)	MPR	66.7 *	50 *	Anemia (6%)

Abbreviations: MPR, major pathologic response; pCR, pathologic complete response; TRAE, treatment-related adverse events; EFS, event-free survival; ALT, alanine aminotransferase; AST, aspartate aminotransferase; PFS, progression-free survival. * denominator = number of patients who underwent surgical resection; ** denominator = intention-to-treat population.

**Table 3:** Selected ongoing clinical trials that included neoadjuvant immunotherapy in resectable NSCLC.

NCT Number	Name	Phase	Stage	Estimated Enrollment	Treatment Arm(s)	Primary Endpoint	Estimated Completion Date
**NCT02938624**	MK3475-223	1	I–II	28	Pembrolizumab at different dose levels	Safety, MPR	April 2021
**NCT02259621**	NA_00092076	2	IB–IIIA	45	Nivolumab + carboplatin + paclitaxel	Safety	January 2023
**NCT03158129**	NEOSTAR	2	IA–IIIA	88	1: Nivolumab + cisplatin + (docetaxel or pemetrexed) 2: Nivolumab + ipilimumab + cisplatin + (docetaxel or pemetrexed)	MPR	July 2022
**NCT03968419**	CANOPY-N	2	IB–IIIA	110	1: Canakinumab 2: Pembrolizumab 3: Canakinumab + pembrolizumab	MPR	March 2023
**NCT04379739**		2	II–IIIA	82	1: Camrelizumab + apatinib 2: Camrelizumab + platinum-based chemotherapy	MPR	December 2026
**NCT04560686**		2	I–IIIB	23	Bintrafusp alfa	MPR	October 2024
**NCT02998528**	CheckMate 816	3	IB–IIIA	350	1: Platinum doublet chemotherapy 2: Nivolumab + platinum doublet chemotherapy 3: Nivolumab + ipilimumab	EFS, pCR	November 2028
**NCT03425643**	KEYNOTE 671	3	II–IIIB	786	1: Pembrolizumab + platinum doublet chemotherapy 2: Placebo + platinum doublet chemotherapy	EFS, OS	June 2026
**NCT03456063**	IMpower 030	3	II–IIIB	453	1: Atezolizumab + platinum doublet chemotherapy 2: Placebo + platinum doublet chemotherapy	EFS	April 2026
**NCT03800134**	AEGEAN	3	II–III	800	1: Durvalumab + platinum-based chemotherapy 2: Placebo + platinum-based chemotherapy	EFS, pCR	April 2024
**NCT04025879**	CheckMate 77T	3	II–IIIB	452	1: Nivolumab + platinum-based doublet chemotherapy 2: Placebo + platinum-based doublet chemotherapy	EFS	September 2024

Abbreviations: MPR, major pathologic response; EFS, event-free survival; pCR, pathologic complete response; OS, overall survival.

**Table 4:** Ongoing clinical trials that included adjuvant immunotherapy in resected NSCLC.

NCT Number	Name	Phase	Stage	Estimated Enrollment	Treatment Arm(s)	Primary Endpoint	Estimated Completion Date
**NCT02273375**	BR.31	3	IB–IIIA	1360	1: Durvalumab 2: Placebo	DFS, DFS in PD-L1 positive patients	January 2023
**NCT02504372**	PEARLS	3	IB–IIIA	1177	1: Pembrolizumab 2: Placebo	DFS	February 2024
**NCT02595944**	ANVIL	3	IB–IIIA	903	1: Nivolumab 2: Observation	OS, DFS, DFS in NSCLC with high PD-L1 expression	July 2024
**NCT03447769**	CANOPY-A	3	IIA–IIIB	1500	1: Canakinumab 2. Placebo	DFS	January 2027
**NCT04385368**	MERMAID-1	3	II–III	332	1: Durvalumab + SoC chemotherapy 2: Placebo + SoC chemotherapy	DFS in MRD+ analysis set	September 2026
**NCT04642469**	MERMAID-2	3	II–III	284	1: Durvalumab 2: Placebo	DFS in PD-L1 ≥ 1% NSCLC	October 2027

Abbreviations: DFS, disease-free survival; PD-L1, programmed death-ligand 1; OS, overall survival; NSCLC, non-small-cell lung cancer; SoC, standard of care; MRD, minimal residual disease.

**Table 5:** Treatment regimens of selected clinical trials that included neoadjuvant immunotherapy in resectable NSCLC.

Trial NCT Number	Trial Name	Neoadjuvant Regimen	Adjuvant Regimen (If Any)
**ChiCTR-OIC-17013726**		2 × siniti 200 mg q3w	SoC/siniti/(siniti + chemo)
**NCT02259621**	NA_00092076	1: 2 × nivo 3 mg/kg q2w 2: 3 × nivo 3 mg/kg q2w + ipi 1 mg/kg 3: 3 × (nivo 360 mg + carbo AUC 5/6 + pacli 175/200 mg) q3w	No
**NCT02818920**	TOP1501	2 × pembro 200 mg q3w	SoC, followed by 4 × pemrbo 200 mg q3w
**NCT02927301**	LCMC3	2 × atezo 1200 mg q3w	atezo 1200 mg ≤ 12 mo
**NCT03030131**	IONESCO	3 × durva 750 mg q2w	No
**NCT03158129**	NEOSTAR	1: 3 × nivo 3 mg/kg q2w 2: 3 × nivo 3 mg/kg q2w + ipi 1 mg/kg 3: 3 × (nivo + cis + doce/peme) q3w 4: 3 × (nivo + cis + doce/peme) q3w + ipi	No
**NCT02572843**	SAKK 16/14	3 × (cis 100 mg/m^2^ + doce 85 mg/m^2^) q3w	2 × durva 750 mg q2w, followed by 26 × durva 750 mg q2w
**NCT02716038**		4 × (atezo 1200 mg + 3 × nab-pacli 100 mg/m^2^ q1w + carbo AUC 5) q3w	No
**NCT03081689**	NADIM	3 × (nivo 360 mg + carbo AUC 6 + pacli 200 mg/m^2^) q3w	nivo 240 mg q2w for 4 mo, followed by nivo 480 mg q4w until mo 12th
**NCT04304248**	NeoTAP01	3 × (toripa 240 mg q3w + 3 × carbo AUC 5 + nab-pacli 260 mg/m^2^/peme 500 mg/m^2^) q3w	toripa ≤ 12 mo
**NCT02938624**	MK3475-223	Cohort 1: pembro 200 mg Cohort 2: 2 × pembro 200 mg q3w Cohort 3: pembro 100 mg	No
**NCT03968419**	CANOPY-N	1: 2 × cana 200 mg q3w 2: 2 × pembro 200 mg q3w 3: 2 × (cana 200 mg + pembro 200 mg) q3w	No
**NCT04379739**		1: (camre 200 mg + apatinib 250 mg) q3w for 2–4 cycles 2: (camre 200 mg + platinum-based chemotherapy) q3w for 2–4 cycles	No
**NCT04560686**		3 × bintrafusp alfa q2w	
**NCT02998528**	CheckMate 816	1: 3 × PBDC q3w 2: 3 × (nivo 360 mg + PBDC) q3w 3: nivo + ipi	No
**NCT03425643**	KEYNOTE 617	1: 4 × (pembro 200 mg + PBDC) q3w 2: 4 × (placebo + PBDC) q3w	13 × pembro 200 mg q3w 13 × placebo q3w
**NCT03456063**	Impower 030	1: 4 × (atezo 1200 mg + PBDC) q3w 2: 4 × (placebo + PBDC) q3w	16 × atezo No
**NCT03800134**	AEGEAN	1: 4 × (durva 1500 mg + PBDC) q3w 2: 4 × (placebo + PBDC) q3w	12 × durva q4w 12 × placebo q4w
**NCT04025879**	CheckMate 77T	1: nivo + PBDC 2: placebo + PBDC	nivo placebo

Abbreviations: siniti, sintilimab; nivo, nivolumab; ipi, ipilimumab; pembro, pembrolizumab; atezo, atezolizumab; durva, durvalumab; toripal, toripalimab; cana, canakinumab; camre, camrelizumab; carbo, carboplatin; pacli, paclitaxel; cis, cisplatin; doce, docetaxel; peme, pemetrexed; PBDC, platinum-based doublet chemotherapy; SoC, standard of care; AUC, area under the curve; q1w, every week; q2w/q3w, once every two/three weeks; mo, months.

**Table 6:** Treatment regimens of selected clinical trials that included adjuvant immunotherapy in resected NSCLC.

Trial NCT Number	Trial Name	Adjuvant Regimen
**NCT00480025**	MACRIT	1: 13 × (recMAGE-A3 + AS15 immunostimulant) 2: 13 × placebo
**NCT02486718**	IMpower 010	1: 4 × cisplatin-based chemotherapy q3w, followed by 16 × atezo 1200 mg q3w 2: 4 × cisplatin-based chemotherapy q3w, followed by best of care
**NCT02273375**	BR.31	1: durva for ≤12 mo 2: placebo for ≤12 mo
**NCT02504372**	Pearls	1: pembro 200 mg q3w for ≤12 mo 2: placebo q3w for ≤12 mo
**NCT02595944**	ANVIL	1: nivo q4w for ≤12 mo 2: observation for 1 year
**NCT03447769**	CANOPY-A	1: 18 × cana 200 mg q3w 2: 18 × placebo q3w
**NCT04385368**	MERMAID-1	1: 12 × (durva 1500 mg + SoC chemotherapy) q3w, followed by 48 × (durva 1500 mg + SoC chemotherapy) q4w 2: 12 × (placebo + SoC chemotherapy) q3w, followed by 48 × (placebo + SoC chemotherapy) q4w
**NCT04642469**	MERMAID-2	1: durva 1500 mg q4w for 24 mo 2: placebo q4w for 24 mo

Abbreviations: nivo, nivolumab; pembro, pembrolizumab; atezo, atezolizumab; durva, durvalumab; cana, canakinumab; SoC, standard of care; q3w/q4w, once every three/four weeks; mo, months.

## References

[1] Sung H., Ferlay J., Siegel R.L., Laversanne M., Soerjomataram I., Jemal A., Bray F. (2021). Global Cancer Statistics 2020: GLOBOCAN Estimates of Incidence and Mortality Worldwide for 36 Cancers in 185 Countries. CA Cancer J. Clin..

[2] Siegel R.L., Miller K.D., Fuchs H.E., Jemal A. (2021). Cancer Statistics, 2021. CA Cancer J. Clin..

[3] Travis W.D., Brambilla E., Nicholson A.G., Yatabe Y., Austin J.H.M., Beasley M.B., Chirieac L.R., Dacic S., Duhig E., Flieder D.B. (2015). The 2015 World Health Organization Classification of Lung Tumors: Impact of Genetic, Clinical and Radiologic Advances since the 2004 Classification. J. Thorac. Oncol..

[4] Kocher F., Hilbe W., Seeber A., Pircher A., Schmid T., Greil R., Auberger J., Nevinny-Stickel M., Sterlacci W., Tzankov A. (2015). Longitudinal analysis of 2293 NSCLC patients: A comprehensive study from the TYROL registry. Lung Cancer..

[5] Howlader N., Noone A.M., Krapcho M., Miller D., Brest A., Yu M., Ruhl J., Tatalovich Z., Mariotto A., Lewis D.R. (2021). SEER Cancer Statistics Review, 1975-2018.

[6] Goldstraw P., Chansky K., Crowley J., Rami-Porta R., Asamura H., Eberhardt W.E.E., Nicholson A.G., Groome P., Mitchell A., Bolejack V. (2016). The IASLC Lung Cancer Staging Project: Proposals for Revision of the TNM Stage Groupings in the Forthcoming (Eighth) Edition of the TNM Classification for Lung Cancer. J. Thorac. Oncol..

[7] Pisters K.M.W., Evans W.K., Azzoli C.G., Kris M.G., Smith C.A., Desch C.E., Somerfield M.R., Brouwers M.C., Darling G., Ellis P.M. (2007). Cancer Care Ontario and American Society of Clinical Oncology adjuvant chemotherapy and adjuvant radiation therapy for stages I-IIIA resectable non small-cell lung cancer guideline. J. Clin. Oncol..

[8] Burdett S., Pignon J.P., Tierney J., Trubodet H., Stewart L., Le Pechoux C., Aupérin A., Le Chevalier T., Stephens R.J., Arriagada R. (2015). Adjuvant chemotherapy for resected early-stage non-small cell lung cancer. Cochrane Database Syst. Rev..

[9] NSCLC Meta-analysis Collaborative Group (2014). Preoperative chemotherapy for non-small-cell lung cancer: A systematic review and meta-analysis of individual participant data. Lancet.

[10] Lim E., Harris G., Patel A., Adachi I., Edmonds L., Song F. (2009). Preoperative versus Postoperative Chemotherapy in Patients with Resectable Non-small Cell Lung Cancer: Systematic Review and Indirect Comparison Meta-Analysis of Randomized Trials. J. Thorac. Oncol..

[11] Gandhi L., Rodríguez-Abreu D., Gadgeel S., Esteban E., Felip E., De Agelis F., Domine M., Clingan P., Hocjmair M.J., Poell S.F. (2018). Pembrolizumab plus Chemotherapy in Metastatic Non–Small-Cell Lung Cancer. N. Engl. J. Med..

[12] Paz-Ares L., Luft A., Vicente D., Tafreshi A., Gümüş M., Mazières J., Hermes B., Çay Şenler F., Csőszi T., Fülöp A. (2018). Pembrolizumab plus Chemotherapy for Squamous Non–Small-Cell Lung Cancer. N. Engl. J. Med..

[13] Gao S., Li N., Gao S., Xue Q., Ying J., Wang S., Tao X., Zhao J., Mao Y., Wang B. (2020). Neoadjuvant PD-1 inhibitor (Sintilimab) in NSCLC. J. Thorac. Oncol..

[14] Forde P.M., Chaft J.E., Smith K.N., Anagnostou V., Cottrell T.R., Hellmann M.D., Zahurak M., Yang S.C., Jones D.R., Broderick S. (2018). Neoadjuvant PD-1 Blockade in Resectable Lung Cancer. N. Engl. J. Med..

[15] Reuss J.E., Anagnostou V., Cottrell T.R., Smith K.N., Verde F., Zahurak M., Lanis M., Murray J.C., Chan H.Y., McCarthy C. (2020). Neoadjuvant nivolumab plus ipilimumab in resectable non-small cell lung cancer. J. Immunother. Cancer.

[16] Tong B.C., Gu L., Wang X., Wigle D.A., Phillips J.D., Harpole D.H., Klapper J.A., Sporn T., Ready N.E., D’Amico T.A. (2021). Perioperative outcomes of pulmonary resection after neoadjuvant pembrolizumab in patients with non-small cell lung cancer. J. Thorac. Cardiovasc. Surg..

[17] Lee J., Chaft J., Nicholas A., Patterson A., Waqar S., Toloza E., Haura E., Raz D., Reckamp K., Merritt R. (2021). PS01.05 Surgical and Clinical Outcomes with Neoadjuvant Atezolizumab in Resectable Stage IB–IIIB NSCLC: LCMC3 Trial Primary Analysis. J. Thorac. Oncol..

[18] Wislez M., Mazieres J., Lavole A., Zalcman G., Carre O., Egenod T., Caliandro R., Gervais R., Geannin G., Molinier O. (2020). 1214O Neoadjuvant durvalumab in resectable non-small cell lung cancer (NSCLC): Preliminary results from a multicenter study (IFCT-1601 IONESCO). Ann. Oncol..

[19] Cascone T., William W.N., Weissferdt A., Leung C.H., Lin H.Y., Pataer A., Godoy M.C.B., Carter B.W., Federico L., Reuben A. (2021). Neoadjuvant nivolumab or nivolumab plus ipilimumab in operable non-small cell lung cancer: The phase 2 randomized NEOSTAR trial. Nat. Med..

[20] Rothschild S.I., Zippelius A., Eboulet E.I., Savic Prince S., Betticher D., Bettini A., Früh M., Joerger M., Lardinoid D., Gelpke H. (2021). SAKK 16/14: Durvalumab in Addition to Neoadjuvant Chemotherapy in Patients with Stage IIIA(N2) Non–Small-Cell Lung Cancer—A Multicenter Single-Arm Phase II Trial. J. Clin. Oncol..

[21] Shu C.A., Gainor J.F., Awad M.M., Chiuzan C., Grigg C.M., Pabani A., Garofano R.F., Stoopler M.B., Cheng S.K., White A. (2020). Neoadjuvant atezolizumab and chemotherapy in patients with resectable non-small-cell lung cancer: An open-label, multicentre, single-arm, phase 2 trial. Lancet Oncol..

[22] Provencio M., Nadal E., Insa A., García-Campelo M.R., Casal-Rubio J., Dómine M., Majem M., Rodríguez-Abreu D., Martínez-Marti A., De Castro Carpenño J. (2020). Neoadjuvant chemotherapy and nivolumab in resectable non-small-cell lung cancer (NADIM): An open-label, multicentre, single-arm, phase 2 trial. Lancet Oncol..

[23] Zhao Z.R., Yang C.P., Chen S., Yu H., Lin Y.B., Lin Y.B., Qi H., Jin J.T., Lian S.S., Wang Y.Z. (2021). Phase 2 trial of neoadjuvant toripalimab with chemotherapy for resectable stage III non-small-cell lung cancer. Oncoimmunology.

[24] Vansteenkiste J.F., Cho B.C., Vanakesa T., De Pas T., Zielinski M., Kim M.S., Jassem J., Yoshimura M., Dahabreh J., Nakayama H. (2016). Efficacy of the MAGE-A3 cancer immunotherapeutic as adjuvant therapy in patients with resected MAGE-A3-positive non-small-cell lung cancer (MAGRIT): A randomised, double-blind, placebo-controlled, phase 3 trial. Lancet Oncol..

[25] Felip E., Altorki N., Zhou C., Csőszi T., Vynnychenko I., Goloborodko O., Luft A., Akopov A., Martinez-Marti A., Kenmotsu H. (2021). Adjuvant atezolizumab after adjuvant chemotherapy in resected stage IB–IIIA non-small-cell lung cancer (IMpower010): A randomised, multicentre, open-label, phase 3 trial. Lancet.

[26] Bar J., Urban D., Ofek E., Ackerstein A., Redinsky I., Golan N., Kamer I., Simansky D., Onn A., Raskin S. (2019). Neoadjuvant pembrolizumab (Pembro) for early stage non-small cell lung cancer (NSCLC): Updated report of a phase I study, MK3475-223. J. Clin. Oncol..

[27] Garrido P., Pujol J.L., Kim E.S., Lee J.M., Tsuboi M., Gómez-Rueda A., Benito A., Moreno N., Gorospe L., Dong T. (2021). Canakinumab with and without pembrolizumab in patients with resectable non-small-cell lung cancer: CANOPY-N study design. Future Oncol..

[28] Neoadjuvant PD-1 Antibody Plus Apatinib or Chemotherapy for Non-small Cell Lung Cancer. https://clinicaltrials.gov/ct2/show/NCT04379739.

[29] Bintrafusp Alfa before Surgery for the Treatment of Untreated Resectable Non-small Cell Lung Cancer. https://clinicaltrials.gov/ct2/show/NCT04560686.

[30] Forde P.M., Chaft J.E., Felip E., Broderick S., Girard N., Awad M.M., Kerr K., Blackwood-Chirchir A., Yang R., Geese W.I. (2017). Checkmate 816: A phase 3, randomized, open-label trial of nivolumab plus ipilimumab vs platinum-doublet chemotherapy as neoadjuvant treatment for early-stage NSCLC. J. Clin. Oncol..

[31] Tsuboi M., Luft A., Ursol G., Kato T., Levchenko E., Eigendorff E., Berard H., Zurawski B., Demedts I., Garassino M.C. (2020). 1235TiP Perioperative pembrolizumab + platinum-based chemotherapy for resectable locally advanced non-small cell lung cancer: The phase III KEYNOTE-671 study. Ann. Oncol..

[32] Peters S., Kim A.W., Solomon B., Gandara D.R., Dziadziuszko R., Brunelli A., Garassino M.C., Reck M., Wang L., To I. (2019). IMpower030: Phase III study evaluating neoadjuvant treatment of resectable stage II-IIIB non-small cell lung cancer (NSCLC) with atezolizumab (atezo) + chemotherapy. Ann. Oncol..

[33] Heymach J., Taube J., Mitsudomi T., Harpole D., Aperghis M., Trani L., Powell M., Dennis P., Reck M. (2019). P1.18-02 The AEGEAN Phase 3 Trial of Neoadjuvant/Adjuvant Durvalumab in Patients with Resectable Stage II/III NSCLC. J. Thorac. Oncol..

[34] Cascone T., Provencio M., Sepesi B., Li S., aanur N., Li S., Spicer J. (2020). Checkmate 77T: A phase III trial of neoadjuvant nivolumab (NIVO) plus chemotherapy (chemo) followed by adjuvant nivo in resectable early-stage NSCLC. J. Clin. Oncol..

[35] Double Blind Placebo Controlled Controlled Study of Adjuvant MEDI4736 In Completely Resected NSCLC. https://clinicaltrials.gov/ct2/show/NCT02273375.

[36] Paz-Ares L., Hasan B., Dafni U., Menis J., Maio E.D., Oselin K., Albert I., Faehling M., Schil P., O’brien M. (2017). A randomized, phase 3 trial with anti-PD-1 monoclonal antibody pembrolizumab (MK-3475) versus placebo for patients with early stage NSCLC after resection and completion of standard adjuvant therapy (EORTC/ETOP 1416-PEARLS). Ann. Oncol..

[37] Chaft J.E., Dahlberg S.E., Khullar O.V., Edelman M.J., Simone C.B., Heymach J., Rudin C.M., Ramalingam S.S. (2018). EA5142 adjuvant nivolumab in resected lung cancers (ANVIL). J. Clin. Oncol..

[38] Garon E.B., Ardizzoni A., Barlesi F., Cho B.C., De Marchi P., Yasushi G., Kowalski D.M., Lu S., Paz-Ares L.G., Spigel D.R. (2020). CANOPY-A: A phase III, multicenter, randomized, double-blind, placebo-controlled trial evaluating canakinumab as adjuvant therapy in patients (pts) with completely resected non-small cell lung cancer (NSCLC). J. Clin. Oncol..

[39] Peters S., Spigel D., Ahn M., Tsuboi M., Chaft J., Harpole D., Goss G., Barlesi F., Abbosh C., Poole L. (2021). P03.03 MERMAID-1: A Phase III Study of Adjuvant Durvalumab plus Chemotherapy in Resected NSCLC Patients with MRD+ Post-Surgery. J. Thorac. Oncol..

[40] Spigel D.R., Peters S., Ahn M.J., Tsuboi M., Chaft J., Harpole D., Barlesi F., Abbosh C., Mann H., May R. (2021). 93TiP MERMAID-2: Phase III study of durvalumab in patients with resected, stage II-III NSCLC who become MRD+ after curative-intent therapy. J. Thorac. Oncol..

[41] Wu Y.L., Tsuboi M., He J., John T., Grohe C., Majem M., Goldman J.W., Laktionv K., Kim S.W., Kabo T. (2020). Osimertinib in Resected EGFR -Mutated Non–Small-Cell Lung Cancer. N. Engl. J. Med..

[42] FDA Approves First Adjuvant Therapy for Most Common Type of Lung Cancer|FDA. https://www.fda.gov/news-events/press-announcements/fda-approves-first-adjuvant-therapy-most-common-type-lung-cancer.

[43] Zhang Y.L., Yuan J.Q., Wang K.F., Fu X.H., Han X.R., Threapleton D., Yang Z.Y., Mao C., Tang J.L. (2016). The prevalence of EGFR mutation in patients with non-small cell lung cancer: A systematic review and meta-analysis. Oncotarget.

[44] FDA Approves Cemiplimab-rwlc for Non-Small Cell Lung Cancer with High PD-L1 Expression|FDA. https://www.fda.gov/drugs/resources-information-approved-drugs/fda-approves-cemiplimab-rwlc-non-small-cell-lung-cancer-high-pd-l1-expression.

[45] FDA Approves Nivolumab Plus Ipilimumab and Chemotherapy for First-Line Treatment of Metastatic NSCLC|FDA. https://www.fda.gov/drugs/resources-information-approved-drugs/fda-approves-nivolumab-plus-ipilimumab-and-chemotherapy-first-line-treatment-metastatic-nsclc.

[46] FDA Approves Atezolizumab for First-Line Treatment of Metastatic NSCLC with High PD-L1 Expression|FDA. https://www.fda.gov/drugs/resources-information-approved-drugs/fda-approves-atezolizumab-first-line-treatment-metastatic-nsclc-high-pd-l1-expression.

[47] FDA Approves Atezolizumab as Adjuvant Treatment for Non-Small Cell Lung Cancer|FDA. https://www.fda.gov/drugs/resources-information-approved-drugs/fda-approves-atezolizumab-adjuvant-treatment-non-small-cell-lung-cancer.

[48] Hellmann M.D., Paz-Ares L., Caro R.B., Zurawski B., Kim S.W., Carcereny Costa E., Park K., Alexandru A., Lupinacci L., de la Mora Jimenez E. (2019). Nivolumab plus Ipilimumab in Advanced Non–Small-Cell Lung Cancer. N. Engl. J. Med..

